# Use of photoimmunoconjugates to characterize ABCB1 in cancer cells

**DOI:** 10.1515/nanoph-2021-0252

**Published:** 2021-07-26

**Authors:** Barry J. Liang, Sabrina Lusvarghi, Suresh V. Ambudkar, Huang-Chiao Huang

**Affiliations:** Fischell Department of Bioengineering, University of Maryland, College Park, MD 20742, USA; and Laboratory of Cell Biology, Center for Cancer Research, National Cancer Institute, National Institutes of Health, Bethesda, MD 20892, USA,; Laboratory of Cell Biology, Center for Cancer Research, National Cancer Institute, National Institutes of Health, Bethesda, MD 20892, USA,; Laboratory of Cell Biology, Center for Cancer Research, National Cancer Institute, National Institutes of Health, Room 2120, Bldg 37, 37 Convent Drive, Bethesda, MD 20892-4256, USA;; Fischell Department of Bioengineering, University of Maryland, 8278 Paint Branch Drive, College Park, MD 20742-5031, USA; and Marlene and Stewart Greenebaum Cancer Center, University of Maryland School of Medicine, Baltimore, MD 21201-1595, USA,

**Keywords:** ABCB1, benzoporphyrin derivative, photodynamic therapy, photoimmunoconjugate, UIC2

## Abstract

Accurate detection of ATP-binding cassette drug transporter ABCB1 expression is imperative for precise identification of drug-resistant tumors. Existing detection methods fail to provide the necessary molecular details regarding the functional state of the transporter. Photo-immunoconjugates are a unique class of antibody–dye conjugates for molecular diagnosis and therapeutic treatment. However, conjugating hydrophobic photosensitizers to hydrophilic antibodies is quite challenging. Here, we devise a photoimmunoconjugate that combines a clinically approved benzoporphyrin derivative (BPD) photosensitizer and the conformational-sensitive UIC2 monoclonal antibody to target functionally active human ABCB1 (i.e., ABCB1 in the inward-open conformation). We show that PEGylation of UIC2 enhances the BPD conjugation efficiency and reduces the amount of non-covalently conjugated BPD molecules by 17%. Size exclusion chromatography effectively separates the different molecular weight species found in the UIC2–BPD sample. The binding of UIC2–BPD to ABCB1 was demonstrated in lipidic nanodiscs and ABCB1-overexpressing triple negative breast cancer (TNBC) cells. UIC2–BPD was found to retain the conformation sensitivity of UIC2, as the addition of ABCB1 modulators increases the antibody reactivity *in vitro*. Thus, the inherent fluorescence capability of BPD can be used to label ABCB1-overexpressing TNBC cells using UIC2–BPD. Our findings provide insight into conjugation of hydrophobic photosensitizers to conformation-sensitive antibodies to target proteins expressed on the surface of cancer cells.

## 1 Introduction

P-glycoprotein (P-gp or ABCB1) is a transmembrane protein of great clinical interest due to its involvement in cancer multidrug resistance (MDR) [1]. It belongs to the ATP-binding cassette (ABC) superfamily of transporters that use ATP hydrolysis to actively transport a wide array of drugs across the cell membrane. During the transport cycle, the ABCB1 transporter adopts an inward-open conformation in which the substrate-binding cavity is exposed, allowing the drug substrate to bind to the transmembrane domain. Subsequent binding of ATP to the nucleotide-binding domain shifts the protein into an inward-closed conformation in which the substrate-binding cavity collapses and the drug is transported out of the cell [2]. A high level of ABCB1 expression has been associated with reduced chemosensitivity in a plethora of cancers such as breast, ovarian, pancreatic, and colorectal cancer [3]. However, a consensus regarding ABCB1 expression and clinical drug resistance has yet to be established due in part to the inconsistency in detecting functional ABCB1 in patient samples [4]. A common method for identifying ABCB1 expression is the reverse transcription polymerase chain reaction (RT-PCR) technique, used to examine the gene transcript or immunohistochemistry to detect protein expression using patient biopsy samples [3]. Inconsistencies observed in ABCB1 detection by different groups may be due to the use of different detecting probes and antibodies, differences in patient selection (i.e., the stage of the disease and whether the patients had received chemotherapy), and the heterogeneity of ABCB1 expression among tumor cells [4]. Additionally, RT-PCR and immunohistochemistry do not provide functional details about the transporters (i.e., the protein conformational state or the efflux activity). Furthermore, longitudinal assessment of the disease is not practical with these methods in pre-clinical research due to the large number of animals needed, or in clinical settings, as repeated biopsies are required. Thus, better strategies are needed to detect and target functional ABCB1.

Fluorescence imaging is an attractive molecular imaging technique to identify and assess ABCB1 activity. Fluorescent substrates of the transporter have been commonly used to image and assess its efflux function in pre-clinical settings. Indocyanine green (ICG) was the first FDA-approved dye used for fluorescence imaging of ABCB1 [5, 6]. However, a statistically non-significant 1.7-fold difference in ICG fluorescence was observed between ABCB1-overexpressing tumors and normal tissues in mouse xenografts [6]. This suggests there is a need for enhanced fluorescence selectivity to label ABCB1 in diseased tissues. The use of fluorescently-labeled antibodies (i.e., antibody–dye conjugates) as imaging agents has been widely investigated due to the advantage of targeting tumor-specific markers with high tumor-to-background ratios [7]. Using an anti-carcinoembryonic antigen (CEA) antibody conjugated with a BM104 fluorophore (SGM-101), Gutowski et al. were able to clearly delineate a CEA-expressing tumor with a tumor-to-background ratio of 3.5 [8]. Currently, SGM-101 is being evaluated in a phase III clinical trial for intra-operative fluorescence visualization of recurrent colorectal cancer (NCT04642924). Similarly, an early clinical study using a folate-fluorescein conjugate (EC17) to target the folate receptor of ovarian cancer showed approximately a 5-fold increase in visualization of malignant lesions in ovarian cancers [9]. Several other antibody–dye conjugates (e.g., Cetuximab–IRDye800, Panitumumab–IRDye800, and Nimotozumab–IRDye800) are also currently in clinical trials for fluorescence-guided surgery of various cancers (NCT03134846, NCT03384238, and NCT04459065, respectively). In addition to fluorescence imaging, some antibody–dye conjugates can also have therapeutic effects; these are known as antibody–photosensitizer conjugates (i.e., photoimmunoconjugates). Photoimmunoconjugates are a unique class of antibody–dye conjugates for molecular diagnosis and therapeutic treatment due to their dual ability to produce reactive molecular oxygen species (e.g., ^1^O_2_, O_2_^•−^, •OH) for biological modulation and to also generate fluorescence signals for imaging [10]. In an exciting development, a Cetuximab-based photoimmunoconjugate (Cetuximab-IRDye700; Akalux^®^) recently received clinical approval from the Ministry of Health, Labor, and Welfare of Japan for treatment of recurrent head and neck cancer [11]. It is currently being investigated as an imaging agent using a clinically approved imaging camera, LIGHT-VISION [12]. Previous attempts to target ABCB1 using photoimmunoconjugates mainly focused on therapeutic outcomes. Mao et al. developed an ABCB1-targeting photoimmunoconjugate that consists of a hydrophilic photosensitizer, IRDye700, and the 15D3 antibody for treatment of drug-resistant tumors [13]. However, research regarding detection of functional ABCB1 expression using photoimmunoconjugates is lacking.

Since the introduction of photoimmunoconjugates by Mew et al. [14], there have been sustained efforts to improve their synthesis and purification. Various photosensitizer conjugation methods have been explored, including carbodiimide coupling [14–16], isothiocyanate conjugation [17, 18], and maleimide chemistry [19]. However, regardless of the conjugation method, it is challenging to covalently link photosensitizers to antibodies due to significant differences in their water solubility. Most photosensitizers are hydrophobic, so they tend to self-aggregate in an aqueous environment. In contrast, antibodies are generally stable in aqueous solutions. Early attempts to facilitate photosensitizer conjugation involved direct structural modification of photosensitizers to incorporate hydrophilicity [20–22]; however, this was found to be cumbersome, with limited benefits, as the final photo-immunoconjugate constructs self-aggregated. Later, a biphasic (organic-aqueous) reaction solvent was introduced to solubilize both the photosensitizers and the antibodies for conjugation. Savellano et al. showed that photosensitizer conjugation in a mixture of 50% dimethyl sulfoxide (DMSO) and 50% aqueous solvent enhances benzoporphyrin derivative (BPD) conjugation to an antibody and minimizes the photosensitizer aggregation during the reaction [23]. Additionally, it was noted that conjugation of polyethylene glycol (PEG) to the antibody is necessary to prevent photoimmunoconjugate aggregation. Using a similar approach, we previously linked BPD molecules on a PEG ylated Cetuximab monoclonal antibody to target cancer cells overexpressing epidermal growth factor receptor (EGFR) [16]. Once the photosensitizers are conjugated, subsequent purification is essential to obtain a pure antibody conjugate. Size-exclusion chromatography is commonly used for purification of photoimmunoconjugates. High-resolution column chromatography methods such as high-performance liquid chromatography (HPLC) or fast protein liquid chromatography (FPLC) are the preferred ways to separate the different molecular weight species of molecules conjugated with different photosensitizers or PEG–antibody ratios [24].

In this study, we sought to devise a photoimmunoconjugate formulation that combines hydrophobic BPD photosensitizers and a conformation-sensitive UIC2 monoclonal antibody to identify ABCB1 expression on triple negative breast cancer (TNBC) cells. TNBC is the most aggressive subtype of breast cancer, characterized by a lack of estrogen, progesterone, and HER2 receptors. It has been demonstrated that ABCB1 expression is ~200% higher in breast cancer tissue compared to normal breast tissue even prior to chemotherapy [25]. Additionally, approximately 50% of TNBC patients develop drug resistance partly due to acquired ABCB1 overexpression during the course of chemotherapy [26]. Therefore, it is imperative to identify and inhibit ABCB1 to prevent the development of drug-resistant TNBC. BPD is a clinically-approved photosensitizer for wet age-related macular degeneration [27]. It is also under clinical investigation for treatment of other malignancies (NCT03033225 and NCT04590664). Recently, a clinical study showed that it is safe and feasible to incorporate light activation of BPD into the current standard of care for breast cancer patients [28]. Hence, BPD is a promising agent to incorporate in a photoimmunoconjugate formulation for diagnosis of ABCB1 expression in breast cancer. In this study, BPD molecules were conjugated to the UIC2 monoclonal antibody to label functionally active ABCB1 (i.e., ABCB1 in the inward-open conformation). UIC2 is a monoclonal antibody that binds to the extracellular region of ABCB1, particularly extracellular loops 1, 3 and 4 [29]. Thus, it allows for labeling of cell surface-expressed ABCB1 without disrupting the plasma membrane. UIC2 is commonly used in a conformation shift assay to identify whether a compound is a substrate of ABCB1 due to its higher reactivity against the functionally active inward-open conformation of ABCB1 [30]. We investigated the effect of UIC2 PEGylation on BPD conjugation efficiency, conjugate recovery, and purity. FPLC purification was used to separate the different molecular species of UIC2–BPD within the heterogeneous conjugated mixture. Our results show that UIC2–BPD conjugate is capable of binding to purified ABCB1 in lipid bilayer nanodiscs and drug-selected VBL-MDA-MB-231 TNBC cells. Furthermore, we demonstrate that UIC2–BPD conjugate can be used to fluorescently label ABCB1 in live TNBC cells, indicating its potential use for fluorescence imaging of tumors expressing this multidrug transporter.

## 2 Experimental section

### 2.1 Chemicals and reagents

Benzoporphyrin derivative (BPD) was purchased from U.S. Pharmacopeia (Rockville, MD). BPD-*N*-hydroxysuccinimidyl ester (BPD-NHS) was synthesized as previously described by us [16]. Poly(ethylene glycol)-*N*-hydroxysuccinimidyl ester (40 kDa, mPEG-NHS) was purchased from Jenkem Technology (Plano, TX). The monoclonal UIC2 antibody was purified from medium containing hybridoma cells as described previously [31]. The monoclonal C219 antibody was purchased from Fujirebio Diagnostics, Inc. (Malvern, PA). The Superdex^®^ 200 Increase 10/300 GL column was purchased from GE Healthcare (Chicago, IL). All other chemicals and reagents were purchased from Thermo Fisher Scientific (Waltham, MA) or Sigma-Aldrich (St. Louis, MO).

### 2.2 UIC2–BPD preparation and characterization

Conjugation of BPD to the monoclonal antibody UIC2 was achieved using carbodiimide chemistry. Briefly, UIC2 antibody (141 kDa; 2 mg/mL) was reacted with mPEG-NHS (40 kDa; 16 mg/mL) at a 1 : 3 or 1 : 8 molar ratio for 18 h at room temperature. The PEGylated UIC2 was then reacted with BPD-NHS at a 1 : 9 molar ratio for 20 h at room temperature before purification using a Zeba^™^ spin desalting column (40 kDa molecular weight cut-off) that was pre-equilibrated with 30% DMSO in phosphate-buffered saline. The resulting UIC2–BPD was concentrated with an Amicon centrifugal filter device (100 kDa molecular weight cut-off). The concentrated product was injected into a Superdex^®^ 200 Increase 10/300 GL column pre-equilibrated with an elution buffer (25 mM *N*-2-hydroxyethylpiperazine-*n*′-2-ethanesulfonic acid buffer pH 7.2 and 150 mM NaCl). Fractions containing the UIC2–BPD conjugates were combined and concentrated using an Amicon centrifugal filter device (100 kDa molecular weight cut-off). The UIC2–BPD purity was assessed by gel fluorescence imaging of BPD after sodium dodecyl sulfate-polyacrylamide gel electrophoresis (SDS-PAGE). The percent of impurity was determined by the fluorescence signal ratio of the free BPD at the bottom of the gel as compared to the total BPD fluorescence signal in the gel. The protein concentration was evaluated with a bicinchoninic acid assay and the BPD concentration was evaluated using UV–Vis spectroscopy using established molar extinction coefficients of 80,500 cm^−1^ M^−1^ and 34,895 cm^−1^ M^−1^ at 435 and 690 nm, respectively [16]. Protein recovery was determined by dividing the protein mass of the purified UIC2–BPD by the mass of UIC2 added initially. The number of BPD molecules per UIC2 was calculated by the molar ratio of BPD to UIC2 after accounting for BPD impurity. BPD conjugation efficiency was determined by dividing the amount of BPD in the purified UIC2–BPD conjugate by the total amount of BPD added initially. The absorbance spectrum and fluorescence characterization (Ex/Em: 435/650–750 nm) of UIC2–BPD were carried out using UV–Vis spectroscopy in a micro-plate reader (Cytation 5, BioTek).

### 2.3 UIC2–BPD binding to ABCB1 reconstituted in lipid bilayer nanodiscs

Human ABCB1 was purified and reconstituted into lipid bilayer nanodiscs as previously described [32]. Native gel electrophoresis was used to determine the binding interaction between UIC2–BPD and ABCB1 lipidic nanodiscs. Briefly, UIC2–BPD or antibody controls (i.e., UIC2 and IgG2a) and ABCB1 lipidic nanodiscs were incubated at 37 °C at a 1 : 1 molar ratio for 5 min. Approximately 3 μg of the sample was loaded into each well of a 4–12% precast NativePAGE^™^ Bis–Tris gel. 1X NativePAGE^™^ blue cathode buffer was added to the electrophoresis chamber to visualize the protein bands. Gel electrophoresis was performed according to the manufacturer’s instructions.

### 2.4 Immunoblotting of ABCB1

ABCB1-overexpressing TNBC VBL-MDA-MB-231 cells and the parental TNBC MDA-MB-231 cells were tested for mycoplasma and maintained in Dulbecco’s Modified Eagle Medium (DMEM) and Roswell Park Memorial Institute Medium (RPMI), respectively. Both growth media were supplemented with 10% (v/v) fetal bovine serum, 100 U/mL penicillin and 100 μg/mL streptomycin. For immunoblotting of ABCB1, approximately 500 K cells were lysed via sonication and a freeze–thaw cycle (4 °C–24 °C) in a lysis buffer (10 mM Tris-Cl pH 8.0, 0.1% Triton X-100, 10 mM MgSO_4_, 2 mM CaCl_2_, 1% aprotinin, 1 mM 4-(2-aminoethyl)benzenesulfonyl fluoride hydrochloride, 2 mM DTT, and 20 μg/mL nuclease). The cell lysates were separated on a 7% precast Tris-acetate protein gel (60 K cells per well) and then transferred to a 0.2 μm nitrocellulose membrane. Protein expression of ABCB1 and glyceraldehyde-3-phosphate dehydrogenase (GAPDH) was labeled using C219 (anti-ABCB1, 1 : 2000 dilution) and GAPDH-6C5 (anti-GAPDH, 1 : 10,000 dilution) primary monoclonal antibodies, respectively. Horseradish peroxidase-conjugated mouse IgG secondary antibody was used at 1 : 10,000 dilution. The blots were visualized by chemiluminescence produced by an ECL Western blotting kit (GE Healthcare).

### 2.5 Selectivity of UIC2–BPD

Selectivity of UIC2–BPD against ABCB1-overexpressing cancer cells was determined using flow cytometry. Briefly, ABCB1 (+) VBL-MDA-MB-231 or ABCB1 (−) MDA-MB-231 cells (200 K cells per tube) were pre-treated with cyclosporine A (CsA, 20 μM) for 5 min at 37 °C to enhance UIC2 antibody binding [33]. Cells were then incubated with UIC2-BPD (6 μg per 200k cells), UIC2 (6 μg per 200k cells), or IgG2a (3 μg per 200k cells) at 37 °C for 30 min before washing with cold phenol red-free Iscove’s Modified Dulbecco’s Medium (IMDM) with 5% (v/v) fetal bovine serum. After washing, fluorescein isothiocyanate (FITC)-labeled secondary antibody (0.25 μg per 100 K cells) was incubated with the cells for 30 min at 37 °C to label the antibody–ABCB1 complex. The labeled cells were washed again and then resuspended in cold phosphate-buffered saline containing 1% bovine serum albumin. Flow cytometry was performed using an FACS CANTO II instrument with BD FACSDiva software to determine the FITC fluorescence (Ex/Em: 488/525 nm). The data were analyzed using FlowJo software.

### 2.6 Fluorescence imaging of UIC2–BPD

Fluorescence imaging of UIC2–BPD was measured using a BioTek LionHeart fluorescence imager. Briefly, VBL-MDA-MB-231 cells (10 K cells per well), cultured in a black-wall flat bottom 96-well plate were pre-treated with cyclosporine A (CsA, 20 μM) for 5 min at 37 °C. UIC2–BPD or UIC2 at varying antibody concentrations (2–8 μg per well) was incubated with the cells for 30 min at 37 °C before washing with phosphate-buffered saline. After washing, FITC-labeled secondary antibody (0.5 μg per well) was added to cells treated with only UIC2 to visualize binding. Cells were imaged with a LionHeart imager using a 10× objective to visualize the BPD (Ex/Em: 445/685 nm) and FITC (Ex/Em: 469/525 nm) signal in the UIC2–BPD and UIC2 treated cells, respectively.

### 2.7 Statistical analysis

Data are presented as mean ± standard deviation (SD) from at least three independent experiments. Statistical analyses were performed using GraphPad Prism (GraphPad Software). Reported p values are two-tailed. One-way ANOVA statistical tests and appropriate post-hoc analyses were applied to avoid type I errors. All data points were included in the analyses.

## 3 Results and discussion

### 3.1 PEGylation of UIC2 antibody increases UIC2–BPD recovery and purity

Conjugation of BPD molecules on the UIC2 antibody was facilitated using carbodiimide chemistry based on an established protocol [16]. The antibody was PEGylated with PEG-NHS (40 kDa) at 1 : 3 and 1 : 8 molar ratios to prevent photoimmunoconjugate aggregation [23]. The conjugation reaction was performed in a 40% DMSO-aqueous environment to minimize BPD-*N*-hydroxysuccinimidyl (NHS) ester aggregation during the reaction. Our data show that increasing the PEGylation ratio from 1 : 3 to 1 : 8 leads to a modest increase of 9% in protein recovery (Table 1). We found that the BPD conjugation efficiency also increased by 8% with the increased PEGylation ratio. This resulted in a final BPD-to-UIC2 molar ratio of 2.0 ± 0.5 and 2.8 ± 0.4 for the 1 : 3 and 1 : 8 PEGylation ratios, respectively (Table 1). Consequently, the amount of non-covalently linked BPD (BPD impurity) was reduced by 17% by increasing the PEGylation ratio.

Previous reports have shown that PEGylation increases the amphiphilicity of conjugated materials [34]. Concerning proteins, increasing amphiphilicity leads to increased stability in a non-aqueous environment. In this study, we interpret the change in protein recovery after increasing the PEGylation ratio to be caused by an increase in antibody stability in a semi-organic environment by increasing the PEG conjugation. This is in accord with a prior study in which protein enzymes were made soluble in organic solvents via PEGylation [35]. Improvement in conjugation efficiency is also likely due to increased antibody solubility, which allows for more lysine residues to be exposed for BPD conjugation via carbodiimide chemistry. While PEGylation stabilizes the antibody for increased BPD conjugation and decreased impurity, it is challenging to separate the PEGylated photoimmunoconjugates and the non-PEGylated photoimmunoconjugates. At a 1 : 3 molar ratio of Cetuximab-to-BPD, Savellano et al. observed photoimmunoconjugate species with different molecular weights within the sample [23]. Similarly, we observed multiple higher molecular weight species of UIC2–BPD after PEG conjugation (Figure S1). This illustrates the problem of non-specific conjugation using carbodiimide chemistry. Interestingly, we also observed BPD conjugation on UIC2 without PEGylation (Figure S1); however, it occurred in only a small fraction of the sample, based on gel electrophoresis. To further separate the different molecular weight species, we proceeded with further purification using high-resolution size exclusion chromatography.

### 3.2 Size-exclusion chromatography separates conjugated UIC2–BPD species

**Figure 1:**
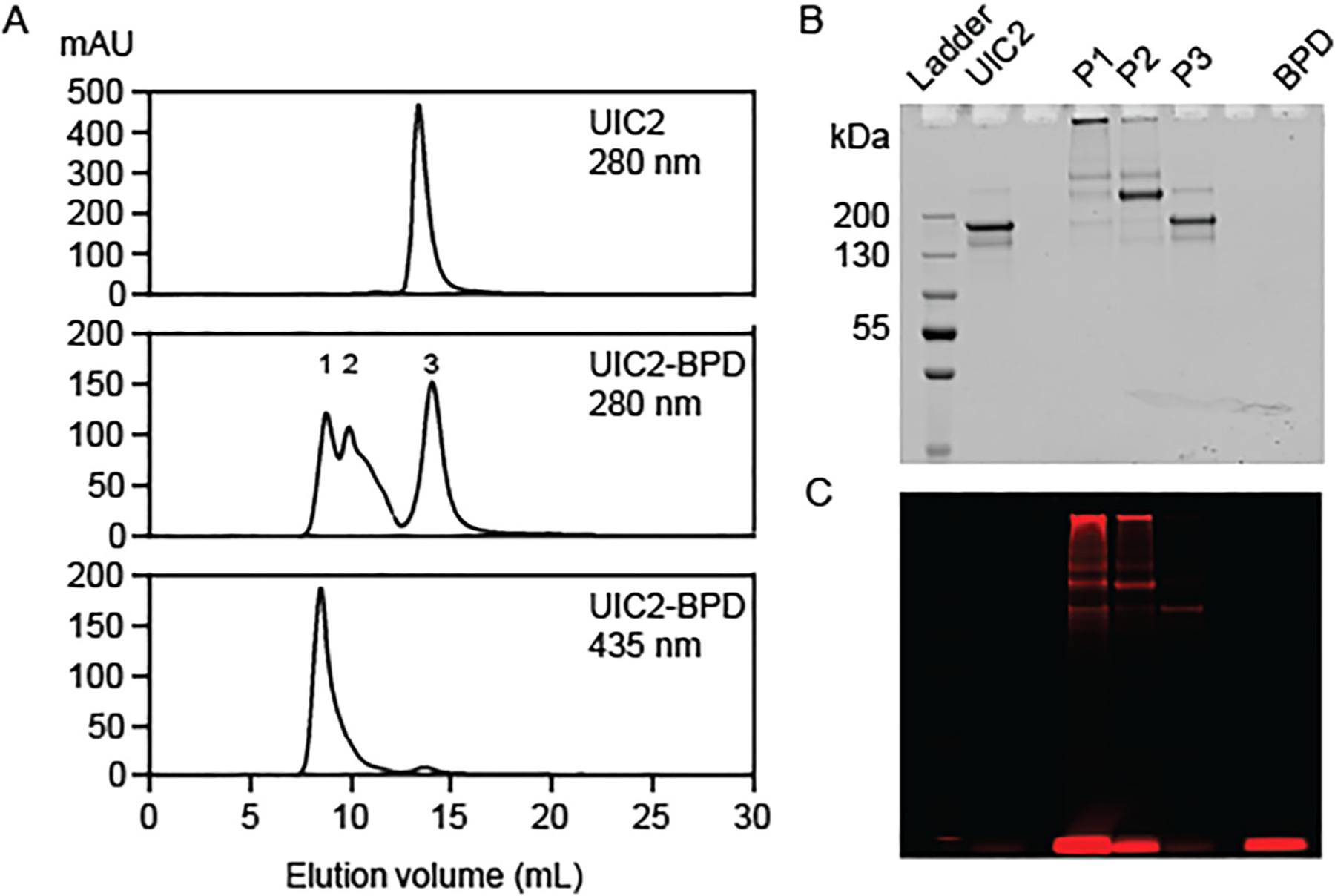
Size exclusion chromatography-FPLC analysis of UIC2–BPD. UIC2–BPD at a BPD : PEG : UIC2 molar ratio of 9 : 8 : 1 was subjected to size exclusion chromatography using a Superdex^®^ 200 increase 10/300 GL column pre-equilibrated with an elution buffer containing 25 mM HEPES pH 7.2 and 150 mM NaCl. (A) FPLC elution profile of UIC2 and UIC2–BPD with UV–Vis detector at 280 and 435 nm. Numbers 1, 2, and 3 indicate the different eluted fractions. (B) Representative InstantBlue^™^ staining of SDS-PAGE for visualization of ladder, unconjugated UIC2, FPLC fractions 1, 2, 3, and free BPD. Higher molecular weight species were observed in the P1 and P2 fractions. (C) Representative fluorescence imaging by SDS-PAGE at 690 nm shows successful conjugation of BPD on UIC2 (top of the gel) and non-covalently conjugated BPD molecules (bottom of the gel).

Size-exclusion chromatography is commonly used for antibody conjugate purification [24]. To separate the different molecular species of UIC2–BPD, we subjected the UIC2–BPD samples to size-exclusion FPLC purification with an ionic aqueous buffer (25 mM HEPES pH 7.2 and 150 mM NaCl) and observed a separation of three major fractions in the 280 nm FPLC chromatogram of the UIC2–BPD sample (Figure 1A, middle panel). The first two fractions (P1 and P2) correspond to the PEGylated UIC2–BPD molecules, as indicated by the protein staining of the high molecular weight species (Figure 1B). Based on the protein staining, we observed that the species in the P1 lane migrated minimally in the gel compared to those in P2. This is likely due to the different degrees of PEGylation in the two fractions. The chromatogram suggests the molecular weight of the protein in the last fraction (P3) of UIC2–BPD is similar to that of the unconjugated UIC2–BPD, as they elute in the same elution volume in FPLC and migrate the same distance in gel electrophoresis (Figure 1A, top and middle panel, and 1B).

The two bottom panels of Figure 1A show the FPLC chromatograms of UIC2–BPD monitored at 280 nm (protein absorbance) and 435 nm (BPD Soret peak), respectively. The overlapping signals from both chromatograms indicate successful BPD conjugation on the UIC2 antibody for the species in P1, which is confirmed by gel electrophoresis (Figure 1B and C). Although we observed BPD fluorescence in the P2 lane (Figure 1C), there is no distinct absorbance signal associated with the P2 elution volume in the 435 nm chromatogram (Figure 1A, bottom panel). We suspect the observed BPD fluorescence in the gel is due to the right tailing of the 435 nm absorbance peak that is associated with P1 (Figure 1A, bottom panel). Additionally, the intensity of the major band in the P2 lane of the protein-stained gel does not correlate with that of the fluorescence gel (Figure 1B and C). Together, our data imply that the observed BPD fluorescence signal in the P2 lane is from the P1 species and the major product in P2 contains minimal BPD conjugation. Due to the lack of sufficient separation, the UIC2–BPD conjugates in the P2 fraction were not characterized.

Based on the molar ratio of BPD-to-UIC2, there are approximately 2.7 ± 0.2 and 0.5 ± 0.1 BPD molecules per UIC2 antibody for the UIC2–BPD conjugates in P1 and P3, respectively (Table 2). We found that FPLC purification further reduced the BPD impurity to 22.5 ± 1.7 and 5.4 ± 0.8% for the UIC2–BPD conjugates in P1 and P3, respectively. While there is approximately 20% non-covalently attached BPD (BPD impurity) in the P1 population, a prior study by Kuimova et al. showed that 20% impurity is within the range for selective targeting using photoimmunoconjugates [36]. Previous studies by us and others have demonstrated that varying the conjugation ratio of BPD molecules per PEGylated Cetuximab antibody molecule allows precise control of BPD quenching and dequenching [16, 23, 37]. Minimal static quenching was observed when only three BPD molecules are conjugated to the PEGylated Cetuximab. Similarly, the UIC2–BPD conjugate only has three BPD molecules conjugated. Thus, we hypothesize that there will be minimal quenching of UIC2–BPD, and the conjugate is readily activated for fluorescence imaging once bound to the cancer cell. One of the preferred subcellular localization sites for free BPD is the mitochondria, where light activation of BPD could induce mitochondrial membrane depolarization [38]. Since BPD is a substrate of ABCB1 [39], the non-covalently-linked BPD molecules could be removed by the ABCB1 expressed on the cancer cells. This implies that a longer photosensitizer-light interval (i.e., the time between photosensitizer administration and light excitation) is needed to avoid mitochondrial damage during fluorescence imaging.

### 3.3 Photophysical characterization of UIC2–BPD

**Figure 2:**
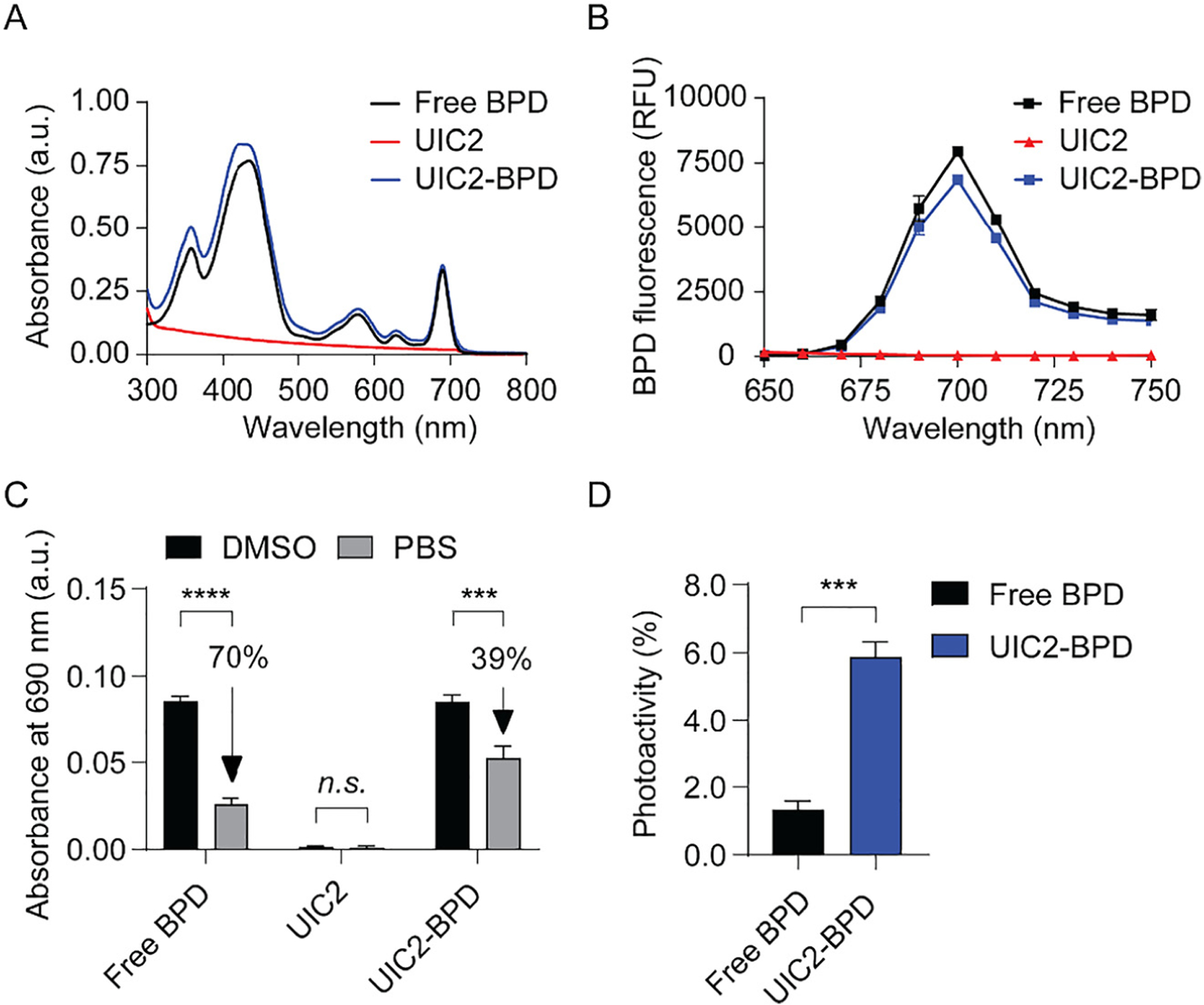
Photophysical characterization of UIC2–BPD. Representative absorbance and fluorescence spectra of UIC2–BPD and respective controls in DMSO. (A) Absorbance spectra of free BPD, UIC2, and UIC2–BPD in DMSO show no alteration to the Soret peak at 435 nm and Q band at 690 nm. (B) Very similar fluorescence spectra of free BPD and UIC2–BPD conjugate in DMSO after light excitation at 435 nm. (C) A comparison of the absorbance value at 690 nm for free BPD, UIC2, and UIC2–BPD. (D) Photoactivity (maximal fluorescence intensity of photosensitizers in phosphate buffered saline divided by that in DMSO) of free BPD and UIC2–BPD. Data presented as mean ± S.D. values from three independent experiments. (*n* = 3, ***P < 0.001, ****P < 0.0001, two-tailed unpaired *t*-test).

Our spectrometric data show that neither the Soret peak (435 nm) nor the Q band (690 nm) of BPD is altered by its conjugation with UIC2, as carbodiimide chemistry does not alter the tetrapyrrole structure of BPD [40] (Figure 2A). This is in agreement with prior studies of BPD conjugation using carbodiimide chemistry [16]. In the clinic, red light (690 nm wavelength) is used for excitation of BPD, as it more deeply (0.1–0.3 cm) penetrates the tissues [41]. This implies that similar light activation parameters of BPD can be used to excite UIC2–BPD. Additionally, light activation of UIC2–BPD at a wavelength of 435 nm produces the same fluorescence spectrum compared to that of BPD (Figure 2B). This suggests that a similar emission bandpass filter of BPD can be used for UIC2–BPD fluorescence visualization. Figure 2C shows a 70% decrease in the absorbance value at 690 nm for free BPD in phosphate-buffered saline compared to that in DMSO. This is due to aggregation of hydrophobic BPD molecules in aqueous solution [42]. The decrease in 690 nm absorbance value of free BPD is consistent with previous findings [16]. In contrast, we observed a 39% decrease in the 690 nm absorbance value for UIC2–BPD in phosphate-buffered saline compared to absorbance at that wavelength in DMSO. We hypothesized that this is due to increased BPD solubility in aqueous solution after protein conjugation. Consequently, conjugation with the UIC2 antibody increased the photoactivity of BPD molecules in aqueous solution by 5% compared to that of free BPD (Figure 2D). This implies that conjugation with UIC2 antibody slightly decreases the static fluorescence quenching of BPD and improves the light activation capability of BPD in physiologically relevant conditions.

### 3.4 UIC2–BPD binding to ABCB1 lipidic nanodiscs

**Figure 3:**
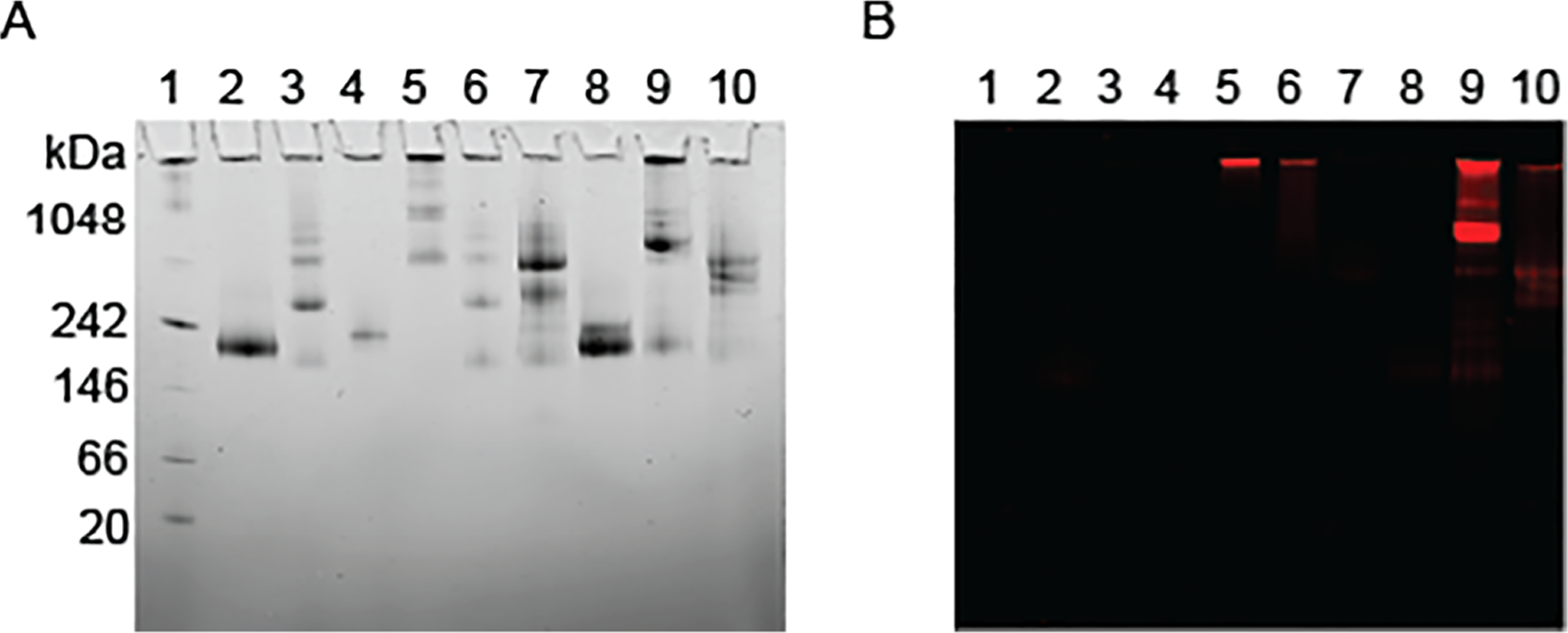
UIC2–BPD binding to purified ABCB1 reconstituted in lipidic nanodiscs. UIC2–BPD was incubated with ABCB1 lipidic nanodiscs at a 1 : 1 molar ratio for 5 min at 37 °C before native gel electrophoresis. (A) Representative cathode dye protein staining was used to visualize the binding interaction between UIC2–BPD and ABCB1 lipidic nanodiscs, and respective controls. Lanes 1–10 correspond with (1) standards, (2) ABCB1 lipidic nanodiscs, (3) UIC2, (4) IgG2a, (5) UIC2–BPD P1, (6) UIC2–BPD P3, (7) ABCB1 lipidic nanodisc + UIC2, (8) ABCB1 lipidic nanodisc + IgG2a, (9) ABCB lipidic nanodisc + UIC2–BPD P1, and (10) ABCB1 lipidic nanodisc + UIC2–BPD P3. (B) Representative fluorescence imaging of the native gel at 690 nm shows BPD fluorescence in the lanes containing UIC2–BPD.

Using native gel electrophoresis, we examined the binding interaction between UIC2–BPD and purified and reconstituted ABCB1 in a lipidic nanodisc model. Lipidic nanodiscs have been proven to be the best platform to study molecular interaction between ABCB1 and other molecules in a membrane environment, as they mimic physiological conditions [32]. Recently, a cryo-EM structure of ABCB1 was solved using ABCB1 lipidic nanodiscs and the fab region of UIC2 [43]. Figure 3A shows the migrated positions of ABCB1 lipidic nanodiscs, UIC2, and IgG2a in lanes 1, 2, and 3, respectively, of a native gel. Due to increased molecular weight resulting from PEGylation, UIC2–BPD P1 had a shorter migration distance in the native gel than unconjugated UIC2 (Figure 3A). In contrast, we observed a similar protein migration pattern when comparing unconjugated UIC2 (lane 3) and UIC2–BPD P3 (lane 6), which implies that both antibody species are of similar molecular weight. This is consistent with our FPLC data, as the unconjugated UIC2 elutes have the same elution volume as UIC2–BPD (Figure 1A). Taken together, these studies suggest minimal PEGylation occurred in the UIC2–BPD P3 population. As expected, the combination of ABCB1 nanodisc and IgG2a in lane 8 did not reduce the migration distance because IgG2a does not bind to ABCB1 (Figure 3A). In contrast, the binding of UIC2–BPD on ABCB1 lipidic nanodiscs is evident in lanes 9 and 10 (Figure 3A). Additionally, fluorescence imaging of the proteins in the native gel reveals BPD fluorescence in the lanes containing UIC2–BPD (Figure 3B). This confirms that the reduced in migration distance of ABCB1 lipidic nanodiscs in the gel is caused by UIC2–BPD binding.

### 3.5 *In vitro* selectivity and conformational sensitivity of UIC2–BPD

**Figure 4:**
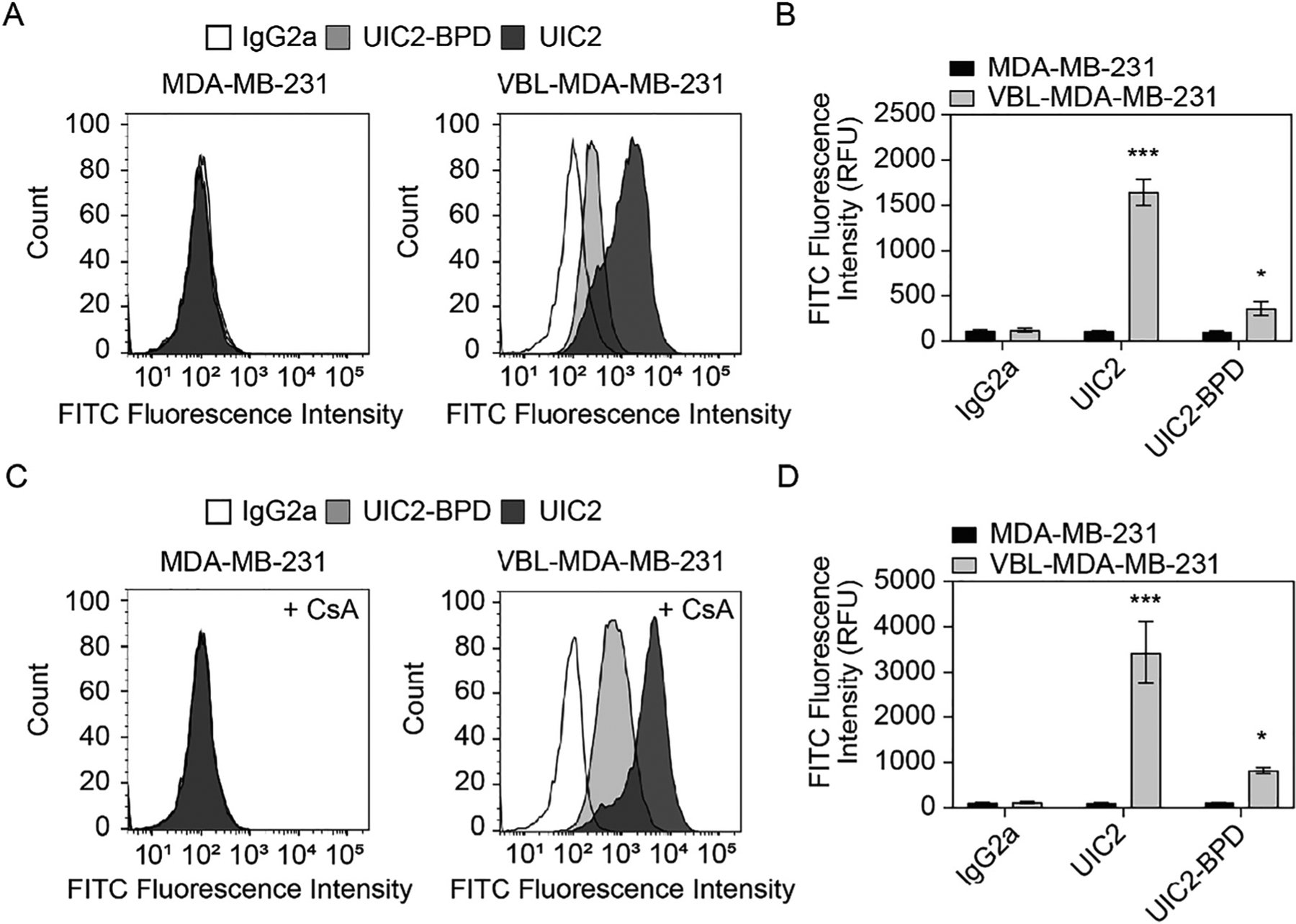
UIC2–BPD binds to VBL-MDA-MB-231 cells. Cells were pre-treated with or without cyclosporine A (CsA, 20 μM) before incubation with IgG2a (3 μg), UIC2 (6 μg), and UIC2–BPD (6 μg) for 30 min at 37 °C. FITC-conjugated secondary antibody was used to label the antibody–ABCB1 complex. Representative histogram of MDA-MB-231 and VBL-MDA-MB-231 cells labeled with FITC secondary antibody (A) without and (C) with the addition of CsA. (B and D) Cellular FITC fluorescence was determined using a flow cytometer with appropriate filter and quantified in FlowJo software. Data presented as mean ± S.D. values from three independent experiments. (*n* = 3, *P < 0.05, ***P < 0.001, one-way ANOVA, Tukey’s post hoc test. Asterisks denote significance compared to the IgG2a group).

We next investigated if UIC2–BPD could selectively bind to ABCB1-overexpressing cells by comparing the antibody–ABCB1 bound complex on ABCB1 (+) drug-resistant VBL-MDA-MB-231 cells and ABCB1 (−) MDA-MB-231 TNBC cells. VBL-MDA-MB-231 is a drug-selected TNBC cell line that overexpresses ABCB1 (Figure S2) and mimics the acquired drug-resistant TNBC cells after chemotherapy [26]. An FITC-labeled secondary antibody is used to identify the UIC2–BPD–ABCB1 bound complex. A representative flow cytometric histogram shows no FITC fluorescence was observed in MDA-MB-231 cells across the different treatments due to the absence of ABCB1 expression on these cells (Figure 4A). Our flow cytometric data show an increase in FITC fluorescence in VBL-MDA-MB-231 cells after treatment with UIC2 or UIC2–BPD but not the isotype IgG2a (Figure 4A). This suggests both UIC2 and UIC2–BPD can bind to ABCB1 expressed on the VBL-MDA-MB-231 cells. However, there is approximately a 4.5-fold difference in FITC fluorescence intensity between UIC2 and UIC2–BPD (Figure 4B). Such a difference implies the binding affinity of UIC2–BPD to ABCB1 is weaker compared to that of UIC2. We hypothesize that this is due to the non-specific antibody modification using carbodiimide chemistry from PEGylation and BPD conjugation. Previous studies by us and others have shown that excess conjugation can compromise the binding reactivity of the antibody [16, 37, 44]. Another explanation for the apparent decrease in FITC fluorescence could be a lack of epitope access for the FITC secondary antibody. The UIC2–BPD conjugates are heavily PEGylated, as indicated by the FPLC and gel electrophoresis data (Figure 1). The presence of the PEG could sterically block the binding of the FITC secondary antibody to the UIC2 antibody. This is similar to “stealth shielding” of nanoparticles by decorating the nanoparticle with PEG to sterically prevent binding of serum protein [45]. While the binding seems to be modestly reduced, the presence of PEG on UIC2–BPD could allow for a longer circulation time in the body after administration.

We further evaluated our hypothesis by examining ABCB1 binding in the UIC2–BPD P3 fraction, which contains less PEGylation and BPD conjugation. Our data show comparable FITC fluorescence in VBL-MDA-MB-231 cells treated with either UIC2 or UIC2–BPD P3 fraction (Figure S3), suggesting a similar affinity of the unconjugated antibody and antibody-conjugate for binding to ABCB1. This is consistent with the reduced binding affinity with increasing antibody modification because the PEG and BPD conjugation in the UIC2–BPD P3 fraction is less than what occurs in the UIC2–BPD P1 fraction.

The addition of selected drug substrates or modulators has been shown to enhance the binding affinity of UIC2 to ABCB1 [30]. UIC2 binds more strongly to the inward-open conformation of ABCB1, which is the functional conformation in which the nucleotide-binding domains are separated and the substrate-binding cavity is open for drug binding [29]. Thus, the ability to distinguish the conformation state of ABCB1 provides details on the functional activity of the transporter. Pre-treatment with cyclosporin A (CsA, an ABCB1 substrate) at 20 μM has been shown to increase the reactivity of UIC2 to ABCB1 [29]. This is consistent with our data using ABCB1-overexpressing VBL-MDA-MB-231 cells (Figure 4C). Similarly, we observed a 2.3-fold increase in FITC fluorescence when the cells were pre-treated with CsA before UIC2–BPD incubation (Figure 4D). These data suggest UIC2–BPD retains conformational sensitivity despite PEGylation and BPD conjugation.

### 3.6 *In vitro* fluorescence labeling of UIC2–BPD

**Figure 5:**
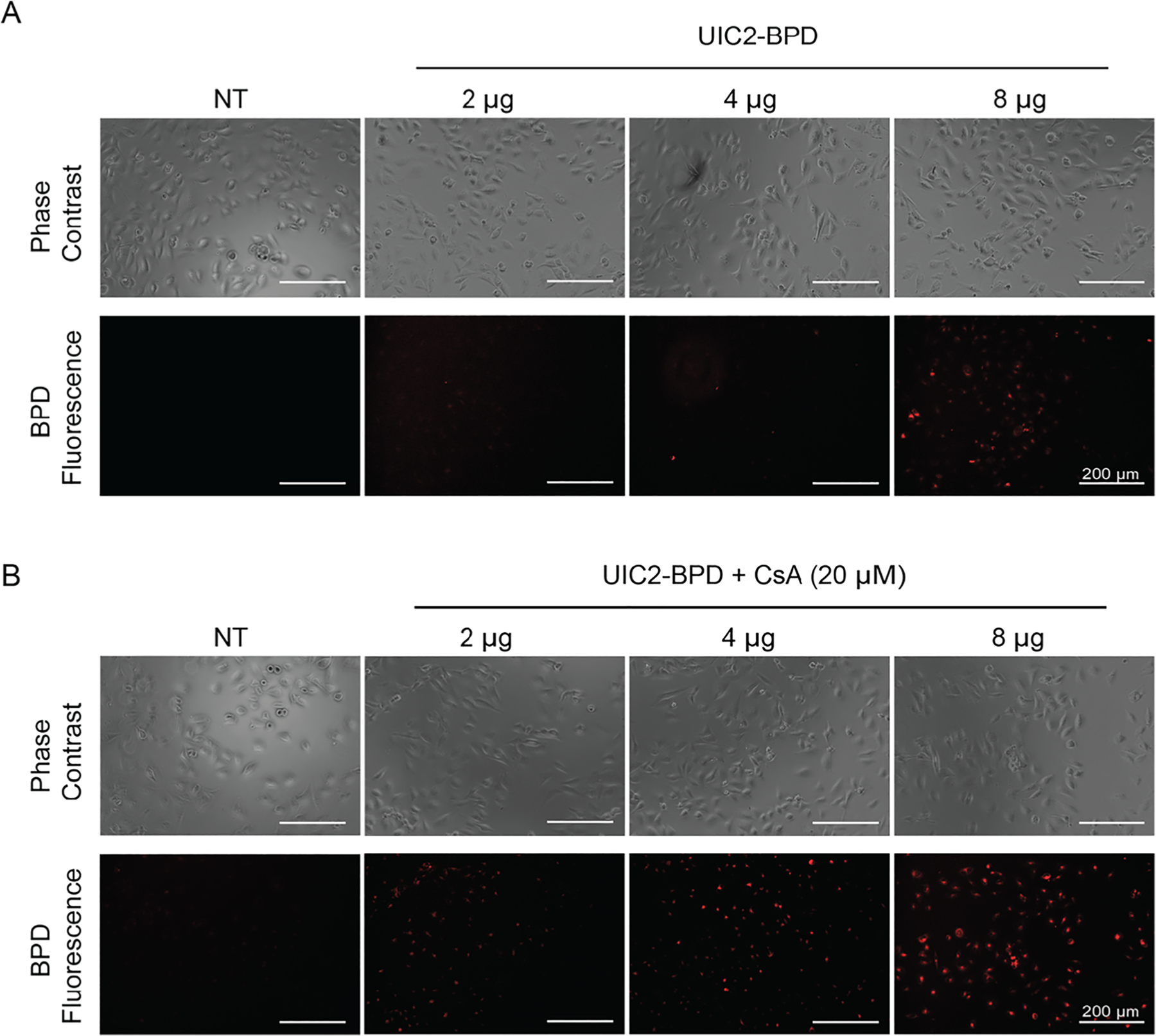
BPD fluorescence imaging of VBL-MDA-MB-231 cells using UIC2–BPD. Representative phase contrast and fluorescence images (Ex/Em: 445/685 nm) of VBL-MDA-MB-231 cells after 30 min of incubation at 37 °C with increasing concentration of UIC2–BPD. (A) BPD fluorescence is the most pronounced at 8 μg without pre-treatment with cyclosporine A (CsA, 20 μM). (B) Pre-treatment with CsA (20 μM) increases the BPD fluorescence in VBL-MDA-MB-231 cells.

Optical molecular labeling of tumors can be applied to cancer treatment in two significant ways. It not only helps researchers to understand the molecular characteristics of the disease but also facilitates identification of the cancerous tissues. One of the unique advantages of photoimmunoconjugates is the ability to leverage both the fluorescence capability of the photosensitizers and the selectivity of the antibody to specifically label cancer cells for fluorescence visualization [46]. We tested if UIC2–BPD could be used for BPD fluorescence imaging of ABCB1-overexpressing TNBC cells. After 30 min of incubation at 37 °C followed by washing to remove unbound conjugates, we observed dose-dependent labeling of the VBL-MDA-MB-231 cells using UIC2–BPD with maximum BPD fluorescence at an 8 μg concentration of the conjugate (Figure 5A). Consistent with the flow cytometric data, the addition of CsA enhances the fluorescence labeling of UIC2–BPD (Figure 5B). These data suggest UIC2–BPD alone can fluorescently label the functionally active ABCB1 molecules that are in the inward-open conformation. Under the same experimental conditions, we observed a similar dose-dependent labeling of VBL-MDA-MB-231 cells using unconjugated UIC2 antibody (Figure S4). Previously, we have shown fluorescence labeling using photoimmunoconjugates is enhanced when conjugated on nanoparticles [16]. Future generations of UIC2–BPD technology could involve the use of the UIC2–BPD photoimmunoconjugate with nanoparticles that are loaded with therapeutic substrate for binding enhancement and disease treatment. The use of photoimmunoconjugates for fluorescence imaging and fluorescence-guided surgery is currently under clinical investigation for a variety of cancers (NCT03134846, NCT03384238, and NCT04459065). In terms of TNBC, there has been active research on incorporating fluorescence-guided surgery for complete resection of tumors. A pre-clinical study by Yano et al. showed that fluorescence-guided surgical removal of TNBC primary tumors using a green fluorescent protein-expressing adenovirus improved overall survival and disease recurrence compared to conventional bright-light surgery [47].

## 4 Conclusion

Conjugation of clinically approved photosensitizers with antibodies for imaging or therapeutic applications remains a challenge due to their high hydrophobicity and the difficulty involved in subsequent conjugate purification. In this work, we report a conjugation protocol to covalently link hydrophobic porphyrin-based BPD photosensitizer molecules to UIC2 antibodies for fluorescence diagnosis of ABCB1-overexpressing TNBC cells. We demonstrate that increasing the PEGylation ratio from 3 : 1 to 8 : 1 (PEG-to-UIC2) improves the BPD conjugation efficiency by 8% and reduces the amount of non-covalently linked BPD molecules by 17%. Our data suggest high-resolution purification using FPLC is necessary to separate the different molecular weight species produced by PEGylation in UIC2–BPD conjugates, which is necessary for accurate fluorescence labeling of ABCB1.

Using purified active ABCB1 reconstituted in lipidic nanodiscs and drug-selected ABCB1-ovexpressing TNBC cells, we show that the resulting UIC2–BPD conjugate can selectively bind to the ABCB1 protein. We observe a conjugation-binding trade-off for UIC2–BPD, in which ABCB1 binding is reduced when using an antibody conjugate with a high ratio of PEGylation and BPD. Nonetheless, our data show effective fluorescence labeling of ABCB1-overexpressing TNBC cells, which can be enhanced in the presence of an ABCB1 substrate. Taken together, our conjugation protocol provides an effective strategy to covalently link hydrophobic BPD molecules on UIC2 antibodies with an acceptable level of unconjugated photosensitizer. This UIC2–BPD conjugation system can be leveraged for fluorescence image-based diagnosis of ABCB1-overexpressing TNBC cells. Further studies are necessary to examine the biodistribution and *in vivo* binding efficacy of the UIC2–BPD conjugate.

## Supplementary Material

Supplementary Figures

## Figures and Tables

**Table 1:** Characterization of UIC2–BPD at different PEG-to-antibody ratios.

Starting reaction stoichiometry (UIC2 : PEG : BPD)	Protein recovery (%)^a^	Conjugation efficiency (%)^b^	BPD impurity (%)^c^	BPD molecules per UIC2^d^
1 : 3 : 9	34.4 ± 6.9	23.2 ± 5.9	47.7 ± 4.4	2.0 ± 0.5
1 : 8 : 9	45.1 ± 5.7	31.4 ± 4.0	30.3 ± 2.9	2.8 ± 0.4

a Protein recovery (%): the protein mass ratio of final UIC2–BPD to that added initially.

b Conjugation efficiency (%): the molar ratio of BPD conjugated to UIC2 to that added initially.

c BPD impurity (%): the fluorescence signal ratio of free BPD to total BPD fluorescence in sample.

d Number of molecules of BPD per UIC2: molar ratio of BPD : UIC2 after accounting for BPD impurity.

**Table 2:** Characterization of purified UIC2–BPD conjugate.

FPLC major fraction	Conjugation efficiency (%)^a^	BPD impurity (%)^b^	BPD per UIC2^c^
1	30.4 ± 2.2	22.5 ± 1.7	2.7 ± 0.2
2	n/a	n/a	n/a
3	5.4 ± 1.5	5.4 ± 0.8	0.5 ± 0.1

a Conjugation efficiency (%): the molar ratio of BPD conjugated to UIC2 to that added initially.

b BPD Impurity (%): the fluorescence signal ratio of free BPD to total BPD fluorescence in sample.

c Number of BPD molecules per UIC2: molar ratio of BPD : UIC2 after accounting for BPD impurity.

## References

[1] Gottesman MM, Fojo T, and Bates SE, “Multidrug resistance in cancer: role of ATP–dependent transporters,” Nat. Rev. Canc, vol. 2, pp. 48–58, 2002. 10.1038/nrc706 11902585

[2] Alam A, Kowal J, Broude E, Roninson I, and Locher KP, “Structural insight into substrate and inhibitor discrimination by human P-glycoprotein,” Science, vol. 363, pp. 753–756, 2019. 30765569 10.1126/science.aav7102 PMC6800160

[3] Robey RW, Pluchino KM, Hall MD, Fojo AT, Bates SE, and Gottesman MM, “Revisiting the role of ABC transporters in multidrug-resistant cancer,” Nat. Rev. Canc, vol. 18, pp. 452–464, 2018. 10.1038/s41568-018-0005-8 PMC6622180 29643473

[4] Leonard GD, Fojo T, and Bates SE, “The role of ABC transporters in clinical practice,” Oncol., vol. 8, pp. 411–424, 2003. 10.1634/theoncologist.8-5-411 14530494

[5] Portnoy E, Gurina M, Magdassi S, and Eyal S, “Evaluation of the near infrared compound indocyanine green as a probe substrate of P-glycoprotein,” Mol. Pharm, vol. 9, pp. 3595–3601, 2012. 23098218 10.1021/mp300472y

[6] Semenenko I, Portnoy E, Aboukaoud M, , “Evaluation of near infrared dyes as markers of P-glycoprotein activity in tumors,” Front. Pharmacol, vol. 7, 2016. 10.3389/fphar.2016.00426. PMC5108765 27895581

[7] Warram JM, de Boer E, Sorace AG, , “Antibody-based imaging strategies for cancer,” Canc. Metastasis Rev, vol. 33, pp. 809–822, 2014. 10.1007/s10555-014-9505-5 PMC4116453 24913898

[8] Gutowski M, Framery B, Boonstra MC, , “SGM-101: an innovative near-infrared dye-antibody conjugate that targets CEA for fluorescence-guided surgery,” Surg. Oncol, vol. 26, pp. 153–162, 2017. 28577721 10.1016/j.suronc.2017.03.002

[9] van Dam GM, Themelis G, Crane LM, , “Intraoperative tumor-specific fluorescence imaging in ovarian cancer by folate receptor-α targeting: first in-human results,” Nat. Med, vol. 17, pp. 1315–1319, 2011. 21926976 10.1038/nm.2472

[10] Kobayashi H and Choyke PL, “Near-Infrared photoimmunotherapy of cancer,” Accounts Chem. Res, vol. 52, pp. 2332–2339, 2019. 10.1021/acs.accounts.9b00273 PMC6704485 31335117

[11] Gomes-da-Silva LC, Kepp O, and Kroemer G, “Regulatory approval of photoimmunotherapy: photodynamic therapy that induces immunogenic cell death,” OncoImmunology, vol. 9, p. 1841393, 2020. 10.1080/2162402X.2020.1841393 PMC7595598 33178498

[12] Inagaki FF, Fujimura D, Furusawa A, , “Fluorescence imaging of tumor-accumulating antibody-IR700 conjugates prior to near-infrared photoimmunotherapy (NIR-PIT) using a commercially available camera designed for indocyanine green,” Mol. Pharm, vol. 18, pp. 1238–1246, 2021. 33502869 10.1021/acs.molpharmaceut.0c01107 PMC9258243

[13] Mao C, Zhao Y, Li F, , “P-glycoprotein targeted and near-infrared light-guided depletion of chemoresistant tumors,” J. Contr. Release, vol. 286, pp. 289–300, 2018. 10.1016/j.jconrel.2018.08.005 PMC6138550 30081143

[14] Mew D, Wat CK, Towers GH, and Levy JG, “Photoimmunotherapy: treatment of animal tumors with tumor-specific monoclonal antibody-hematoporphyrin conjugates,” J. Immunol, vol. 130, pp. 1473–1477, 1983. 6185591

[15] Mitsunaga M, Ogawa M, Kosaka N, , “Cancer cell-selective in vivo near infrared photoimmunotherapy targeting specific membrane molecules,” Nat. Med, vol. 17, pp. 1685–1691, 2011. 22057348 10.1038/nm.2554 PMC3233641

[16] Liang BJ, Pigula M, Baglo Y, Najafali D, Hasan T, and Huang H-C, “Breaking the selectivity-uptake trade-off of photoimmunoconjugates with nanoliposomal irinotecan for synergistic multi-tier cancer targeting,” J. Nanobiotechnol, vol. 18, p. 1, 2020. 10.1186/s12951-019-0560-5 PMC6939330 31898555

[17] Hudson R, Carcenac M, Smith K, , “The development and characterisation of porphyrin isothiocyanate–monoclonal antibody conjugates for photoimmunotherapy,” Br. J. Canc, vol. 92, pp. 1442–1449, 2005. 10.1038/sj.bjc.6602517 PMC2362018 15812551

[18] Malatesti N, Smith K, Savoie H, Greenman J, and Boyle RW, “Synthesis and in vitro investigation of cationic 5,15-diphenyl porphyrin-monoclonal antibody conjugates as targeted photodynamic sensitisers,” Int. J. Oncol, vol. 28, pp. 1561–1569, 2006. 16685457

[19] Alonso CMA, Palumbo A, Bullous AJ, Pretto F, Neri D, and Boyle RW, “Site-specific and stoichiometric conjugation of cationic porphyrins to antiangiogenic monoclonal antibodies,” Bioconjugate Chem., vol. 21, pp. 302–313, 2010. 10.1021/bc9003537 20073477

[20] Wagner RW, Lindsey JS, Turowska-Tyrk I, and Scheidt WR, “Synthesis of porphyrins tailored with eight facially-encumbering groups. An approach to solid-state light-harvesting complexes,” Tetrahedron, vol. 50, pp. 11097–11112, 1994.

[21] Ahmed S, Davoust E, Savoie H, Boa AN, and Boyle RW, “Thioglycosylated cationic porphyrins—convenient synthesis and photodynamic activity in vitro,” Tetrahedron Lett., vol. 45, pp. 6045–6047, 2004.

[22] Borbas KE, Mroz P, Hamblin MR, and Lindsey JS, “Bioconjugatable porphyrins bearing a compact swallowtail motif for water solubility,” Bioconjugate Chem., vol. 17, pp. 638–653, 2006. 10.1021/bc050337w PMC3072562 16704201

[23] Savellano MD and Hasan T, “Targeting cells that overexpress the epidermal growth factor receptor with polyethylene glycolated BPD verteporfin photosensitizer immunoconjugates,” Photochem. Photobiol, vol. 77, pp. 431–439, 2003. 12733655 10.1562/0031-8655(2003)077<0431:tctote>2.0.co;2

[24] Hong P, Koza S, and Bouvier ES, “Size-exclusion chromatography for the analysis of protein biotherapeutics and their aggregates,” J. Liq. Chromatogr. Relat. Technol, vol. 35, pp. 2923–2950, 2012. 23378719 10.1080/10826076.2012.743724 PMC3556795

[25] Nedeljković M, Tanić N, Prvanović M, Milovanović Z, and Tanić N, “Friend or foe: ABCG2, ABCC1 and ABCB1 expression in triple-negative breast cancer,” Breast Cancer, vol. 28, pp. 727–736, 2021. 33420675 10.1007/s12282-020-01210-z

[26] Foulkes WD, Smith IE, and Reis-Filho JS, “Triple-negative breast cancer,” N. Engl. J. Med, vol. 363, pp. 1938–1948, 2010. 21067385 10.1056/NEJMra1001389

[27] Schmidt-Erfurth U, Hasan T, Gragoudas E, Michaud N, Flotte TJ, and Birngruber R, “Vascular targeting in photodynamic occlusion of subretinal vessels,” Ophthalmology, vol. 101, pp. 1953–1961, 1994. 7997334 10.1016/s0161-6420(13)31079-3

[28] Banerjee SM, El-Sheikh S, Malhotra A, , “Photodynamic therapy in primary breast cancer,” J. Clin. Med, vol. 9, p. 483, 2020. 10.3390/jcm9020483 PMC7074474 32050675

[29] Vahedi S, Lusvarghi S, Pluchino K, , “Mapping discontinuous epitopes for MRK-16, UIC2 and 4E3 antibodies to extracellular loops 1 and 4 of human P-glycoprotein,” Sci. Rep, vol. 8, p. 12716, 2018. 10.1038/s41598-018-30984-8 PMC6109178 30143707

[30] Mechetner EB and Roninson IB, “Efficient inhibition of P-glycoprotein-mediated multidrug resistance with a monoclonal antibody,” Proc. Natl. Acad. Sci. U. S. A, vol. 89, pp. 5824–5828, 1992. 1352877 10.1073/pnas.89.13.5824 PMC402110

[31] Frank GA, Shukla S, Rao P, , “Cryo-EM analysis of the conformational landscape of human P-glycoprotein (ABCB1) during its catalytic cycle,” Mol. Pharmacol, vol. 90, pp. 35–41, 2016. 27190212 10.1124/mol.116.104190 PMC4931865

[32] Nandigama K, Lusvarghi S, Shukla S, and Ambudkar SV, “Large-scale purification of functional human P-glycoprotein (ABCB1),” Protein Expr. Purif, vol. 159, pp. 60–68, 2019. 30851394 10.1016/j.pep.2019.03.002 PMC6600825

[33] Goda K, Fenyvesi F, Bacsó Z, , “Complete inhibition of P-glycoprotein by simultaneous treatment with a distinct class of modulators and the UIC2 monoclonal antibody,” J. Pharmacol. Exp. Therapeut, vol. 320, pp. 81–88, 2007. 10.1124/jpet.106.110155 17050779

[34] Stepankova V, Bidmanova S, Koudelakova T, Prokop Z, Chaloupkova R, and Damborsky J, “Strategies for stabilization of enzymes in organic solvents,” ACS Catal., vol. 3, pp. 2823–2836, 2013.

[35] Inada Y, Takahashi K, Yoshimoto T, Ajima A, Matsushima A, and Damborsky J, “Application of polyethylene glycolmodified enzymes in biotechnological processes: organic solvent-soluble enzymes,” Trends Biotechnol., vol. 4, pp. 190–194, 1986.

[36] Kuimova MK, Bhatti M, Deonarain M, , “Fluorescence characterisation of multiply-loaded anti-HER2 single chain Fvphotosensitizer conjugates suitable for photodynamic therapy,” Photochem. Photobiol. Sci, vol. 6, pp. 933–939, 2007. 17721591 10.1039/b708320c

[37] Spring BQ, Abu-Yousif AO, Palanisami A, , “Selective treatment and monitoring of disseminated cancer micrometastases in vivo using dual-function, activatable immunoconjugates,” Proc. Natl. Acad. Sci. U. S. A, vol. 111, pp. E933–E942, 2014. 24572574 10.1073/pnas.1319493111 PMC3956156

[38] Inglut CT, Baglo Y, Liang BJ, , “Systematic evaluation of light-activatable biohybrids for anti-glioma photodynamic therapy,” J. Clin. Med, vol. 8, p. 1269, 2019. 10.3390/jcm8091269 PMC6780262 31438568

[39] Baglo Y, Liang BJ, Robey RW, Ambudkar SV, Gottesman MM, and Huang H-C, “Porphyrin-lipid assemblies and nanovesicles overcome ABC transporter-mediated photodynamic therapy resistance in cancer cells,” Canc. Lett, vol. 457, pp. 110–118, 2019. 10.1016/j.canlet.2019.04.037 PMC6690745 31071369

[40] Abrahamse H and Hamblin MR, “New photosensitizers for photodynamic therapy,” Biochem. J, vol. 473, pp. 347–364, 2016. 26862179 10.1042/BJ20150942 PMC4811612

[41] Yun SH and Kwok SJJ, “Light in diagnosis, therapy and surgery,” Nat. Biomed. Eng, vol. 1, 2017, Art no. 0008. 10.1038/s41551-016-0008 PMC5476943 28649464

[42] Chen B, Pogue BW, and Hasan T, “Liposomal delivery of photosensitising agents,” Expet Opin. Drug Deliv, vol. 2, pp. 477–487, 2005. 10.1517/17425247.2.3.477 16296769

[43] Nosol K, Romane K, Irobalieva RN, , “Cryo-EM structures reveal distinct mechanisms of inhibition of the human multidrug transporter ABCB1,” Proc. Natl. Acad. Sci. U. S. A, vol. 117, pp. 26245–26253, 2020. 33020312 10.1073/pnas.2010264117 PMC7585025

[44] Huang HC, Pigula M, Fang Y, and Hasan T, “Immobilization of photo-immunoconjugates on nanoparticles leads to enhanced light-activated biological effects,” Small, vol. 14, 2018, Art no. e1800236. 10.1002/smll.201800236 PMC6312758 29962083

[45] Li S-D and Huang L, “Stealth nanoparticles: high density but sheddable PEG is a key for tumor targeting,” J. Contr. Release, vol. 145, pp. 178–181, 2010. 10.1016/j.jconrel.2010.03.016 PMC2902652 20338200

[46] Tang Q, Nagaya T, Liu Y, , “Real-time monitoring of microdistribution of antibody-photon absorber conjugates during photoimmunotherapy in vivo,” J. Contr. Release, vol. 260, pp. 154–163, 2017. 10.1016/j.jconrel.2017.06.004 PMC5726775 28601576

[47] Yano S, Takehara K, Miwa S, Kishimoto H, Tazawa H, , “Fluorescence-guided surgery of a highly-metastatic variant of human triple-negative breast cancer targeted with a cancer-specific GFP adenovirus prevents recurrence,” Oncotarget, vol. 7, pp. 75635–75647, 2016. 27689331 10.18632/oncotarget.12314 PMC5342766

