# Supplementary material for: Use of photoimmunoconjugates to characterize ABCB1 in cancer cells
Source: Nanophotonics. Author manuscript; Available in PMC 2022 Jan 20. (PMC8773461; doi:10.1515/nanoph-2021-0252)
Supplement: Supplementary Figures [file NIHMS1737455-supplement-Supplementary_Figures.pdf]

Supplementary Figures

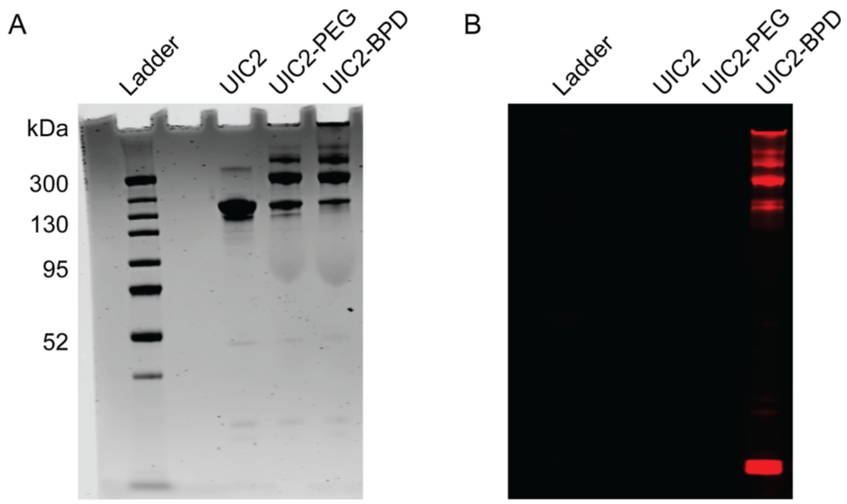

Figure S1: Sodium dodecyl sulfate polyacrylamide gel electrophoresis of UIC2-BPD conjugate. UIC2-BPD at a BPD: PEG: UIC2 molar ratio of 9:8:1 was subjected to SDS-PAGE before FPLC purification. (A) Representative InstantBlue^{TM} staining of SDS-PAGE for visualization of ladder, unconjugated UIC2, UIC2-PEG at 8:1 (PEG:UIC2), and UIC2-BPD at 9:8:1 (BPD:PEG:UIC2) (B) Representative fluorescence imaging by SDS-PAGE at 690 nm shows conjugation of BPD to UIC2 (top of the gel) and non-covalently conjugated BPD molecules (bottom of the gel).

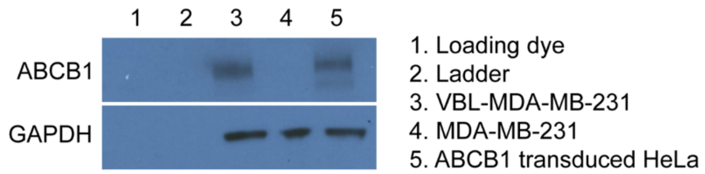

Figure S2: Immunoblotting of ABCB1 expressed in VBL-MDA-MB-231, MDA-MB-231, and ABCB1-transduced HeLa cells. Cell lysates (60 K cells per well) were separated with gel electrophoresis before being transferred to a 0.2 μm nitrocellulose membrane. ABCB1 and GAPDH were labeled with C219 (anti-ABCB1, 1:2000 dilution) and GAPDH-6C5 (anti-GAPDH, 1:10,000 dilution), respectively.

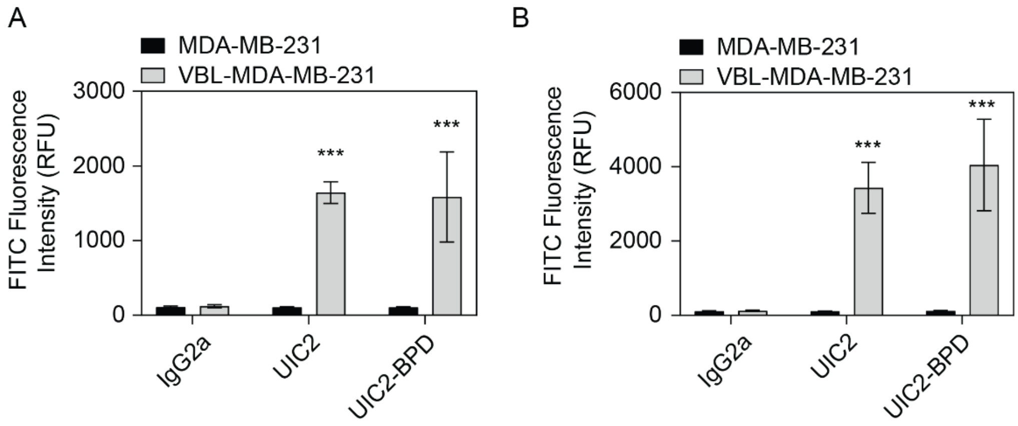

Figure S3: UIC2-BPD P3 binds to VBL-MDA-MB-231 cells. Cells were pre-treated with or without cyclosporine A (20 μM) before UIC2-BPD incubation. FITC-conjugated secondary antibody was used to label antibody-ABCB1 complex. Cellular FITC fluorescence of VBL-MDA-MB-231 cells (A) in the absence and (B) presence of cyclosporine A was determined using a flow cytometer with appropriate filter and quantified in FlowJo software. Data presented are the mean ± S.D. values from three independent experiments (n=3, ***P < 0.001, one-way ANOVA, Tukey's post hoc test. Asterisks denote significance compared to the IgG2a group).

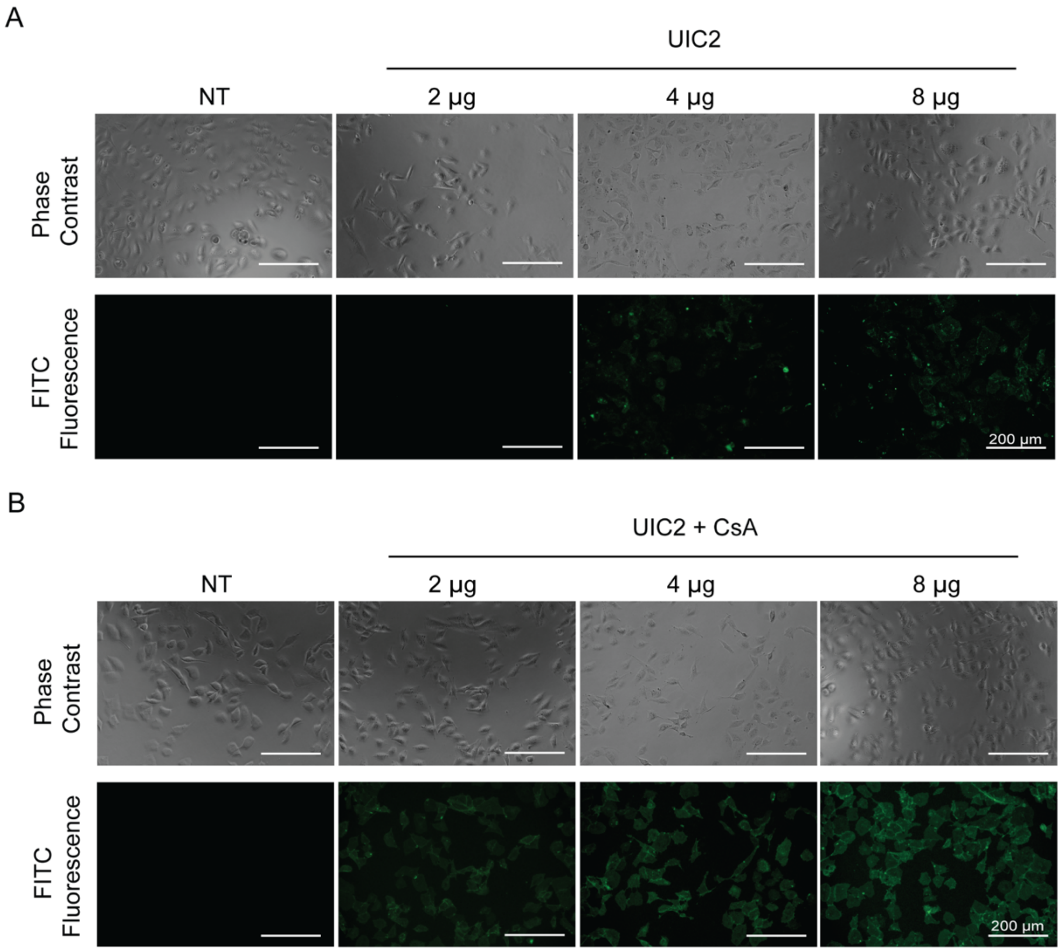

Figure S4: UIC2 labeling of VBL-MDA-MB-231 cells. Cells were pre-treated with or without cyclosporine A (20 µM) before incubation with UIC2 at increasing concentrations. FITC-conjugated secondary antibody was used to label antibody-ABCB1 complex. Representative phase contrast and FITC fluorescence images (Ex/Em: 469/525 nm) of VBL-MDA-MB-231 cells (A) in the absence and (B) presence of cyclosporine A.
